# Profiles of Problematic Internet Use in Bullying and Cyberbullying among Adolescents

**DOI:** 10.3390/ijerph17197041

**Published:** 2020-09-26

**Authors:** Inmaculada Méndez, Ana Belén Jorquera, Cecilia Ruiz Esteban, José Manuel García-Fernández

**Affiliations:** 1 Evolutionary and Educational Psychology, Faculty of Psychology, University of Murcia, 30100 Murcia, Spain; inmamendez@um.es (I.M.); anabelen.jorquera@um.es (A.B.J.); 2 Department of Developmental Psychology and Didactics, University of Alicante, 03080 Alicante, Spain

**Keywords:** bullying, cyberbullying, emotional, adolescence, emotional adjustment

## Abstract

The rise of technology has increased risks such as problematic internet use or cyberbullying. Data show that there is problematic use of the internet, which has important repercussions academically, personally, socially and for health. The objective of this study was to identify different profiles that vary according to intra- and interpersonal conflicts related to internet use. In addition, this study aimed to examine whether there are significant differences in bullying and cyberbullying among adolescents with a conflict related to internet use. The study participants were 810 students of Compulsory Secondary Education (*M* = 13.99, *SD* = 1.32). The Questionnaire on School Violence and the Questionnaire of Experiences Related to Internet was use. The latent profile analysis identified four different types of conflicts related to internet use: (a) high levels in intra- and interpersonal conflicts; (b) low levels intra- and interpersonal conflicts; (c) moderate intra- and interpersonal conflicts and (d) very high levels in intra- and interpersonal conflicts. The results of the study indicated that there were significant differences in the manifestations of school violence between the profiles. This study assists in educational programs to prevent conflicts related to internet use and school violence through emotional adjustment.

## 1. Introduction

The internet allows quick access to information and allows us to maintain contact through social networks and email; however, excessive and persistent recreational use can lead to problems. A person who presents compulsive or problematic use spends excessive time connected to the internet, typically for recreational purposes, which can lead them to neglect other important areas of their daily life, such as work or studies, social relationships, food, or rest [1,2,3,4].

Specifically, the person loses control and becomes dependent due to the abusive use of the internet, which is linked to the fact that it initially presents a pleasant effect (positive reinforcer) at the same time that it reduces emotional tension (negative reinforcer). Given that problematic internet use has been associated with an imbalance in emotional regulation [5,6], the person typically enters a circle in which they seek to alleviate their emotional discomfort through abusive use of the internet, which helps them to minimize these emotions [2,4,6,7,8,9].

Compulsive or problematic use of the internet is currently classified as part of behavioral addictions because this behavior presents characteristics similar to substance addictions [3,4,10,11]. The Diagnostic and Statistical Manual of Mental Disorders (DSM-5) [12] collects substance-free addictions but has not collected presentely internet addiction despite existing literature on the associated risks [4,7,13]. Scientific evidence indicates that problematic internet use is often associated with other psychological problems, such as depression, irritability, anxiety, social phobia, and drug use [4,9,13,14] or may even be the secondary manifestation of another major addiction, such as sex addiction [2,4,14,15].

Social media present positive aspects since they encourages the use of communication and establishes interpersonal relationships [7,8,16,17,18]; however, negative aspects, such as interpersonal conflicts may also appear, i.e., between those surrounding the individual (for example, misaligned behaviors such as presenting an imbalance between what is done and what is said), as well as intrapersonal conflicts, i.e., those that involve the individual in relation to the activity itself (for example, a lack of emotional self-regulation) [7,19,20]. Digital interpersonal relationships imply a change in spatial and temporal perception, creating a sense of immediacy of events and the acceleration of processes [8,16]. Coupled with the fact that the user does not need to be identified (for example, in chat rooms, the identity can be altered), this can produce a bespoke idealized character, which favors online social interaction versus face-to-face verbal communication [4,21,22,23]. Therefore, cybercommunication can generate intrapersonal and interpersonal conflicts that differ from those existing in non-virtual communication. Cybercommunication implies a permanent connection as well as new styles of communication, the user being able to disconnect whenever he wishes. Intrapersonal conflicts may even appear due to the need, for example, to use the internet to escape from the routine or as a way to escape from anxiety [7,8,16].

The use or compulsive internet has been increasing in recent years, in particular among the youngest in the population [24,25]. Specifically, in Spain, a report presented by the Government Delegation for the National Plan on Drugs [24] showed that 20% of the population of schoolchildren between the ages of 14 and 18 made compulsive use of the internet. This is a number of adolescents who are at high risk for compulsive use of the internet in our country.

Within the adolescent stage, the search for identity plays a relevant role in the use of the internet, and thus a confusion of identity was associated with problematic use of the internet [26]. Teens seek immediate satisfaction through virtual communication, and the feedback received by teens can be either harmful or enriching [21]. Virtual communication can involve the risks of anonymity, and it is difficult to differentiate the public from the private, causing the person to lose control of what they share on the internet. Therefore, there is a clear association between problematic internet use, social media membership, and the perception of privacy on the internet.

Publicly exposing information makes the individual vulnerable to cyberbullying as sharing a physical location increases the likelihood of real-life harassment [27,28]. Thereby, cyberbullying is more likely among young people who share a technological culture, with positive attitudes and beliefs regarding the intensive use of the internet assuming an extension of their personal biography and problematic interpersonal relationships, such as bullying [27,29,30,31,32,33,34,35]. In this regard, studies have shown that problematic use of the internet was linked to greater implications in the different manifestations of school violence (bullying and cyberbullying) [36,37] both in the role of victim and as an aggressor in the face of those not involved [38,39,40,41,42].

Interpersonal conflicts derived from compulsive internet use play an important role in cyberbullying [40,41]. A deficiency of interpersonal factors that can contribute to social relations of rejection or social exclusion [41]. Likewise, it has been found that intrapersonal conflicts are related to cyberbullying roles [40].

Previous studies have examined clusters or groups for problematic Internet use with the CERI scale, finding low, moderate or high scores among young people aged 11 to 25 years [22,43]. The most maladaptive profile of the students was the one that he made greater use of the Internet, he related to people through the Internet, even abandoning his academic obligations, which caused a decrease in academic performance and even had serious subsequent consequences [43].

For all the above, the objective of this study was to identify different profiles that vary according to intrapersonal and interpersonal conflicts related to internet use. In addition, this study aimed to examine whether there are significant differences in bullying and cyberbullying among adolescents with a conflict related to internet use.

The main hypotheses are: (1) there are different profiles of problematic conflicts related to internet use (intrapersonal and interpersonal) among adolescents; (2) adolescents with conflict related to internet use will be more involved in problems of school violence (bullying and cyberbullying).

## 2. Materials and Methods 

### 2.1. Design and Participants

We performed a cross-sectional study in the Region of Murcia. First, a school center was randomly selected from each of the geographical areas of the Region of Murcia. The class group participating in each course was then randomly selected. We recruited 1021 students from different educational centers for the study; however, 211 were excluded because they did not have informed consent, they were not present on the day of administration of the instruments, or because their evaluation instruments were incomplete (not fully completed). Therefore, a total of 810 students (52.2% girls and 47.8% boys) took part in this study from Secondary Compulsory Education of the Region of Murcia, Spain. The ages ranged from 12 to 16 years old (*M* = 13.99, *SD* = 1.32); thus, 77.9% had not repeated a course a school year. 4.8% were born outside of Spain. The distribution was homogeneous in terms of sex and age (χ2 = 4.33, *p* = 0.50). The socio-economic level of the different areas and schools was medium (urban and rural areas). The ethnic composition was mostly Spanish, followed by Hipanos, other Europeans, North Africans and Asians. The parents had mainly secondary education, followed by primary and to a lesser extent university

### 2.2. Instruments

To measure the sociodemographic characteristics and academic issues, the following variables were assessed: sex (male/female), age, country of birth, course repetition (yes/no), and type of the school (public/private/semi-private).

The perceptions of school violence occurring in school settings were evaluated through the Psychometric Properties of School Violence Questionnaire-Revised [44]. This questionnaire consists of 31 items, and the responses are recorded on a rating scale (1 = never, 5 = always). The questionnaire consisted of eight factors of the different manifestations of school violence: violence of teachers towards students (VTS), physical indirect violence by students (VPI), verbal violence among students (VVS), physical direct violence between students (VPD), verbal violence of students towards teachers (VVT), social exclusion (SE), disruptive behavior in the classroom (DB), and violence through information and communication technology (VICT). The Cronbach’s α coefficients were shown to range from 0.67–0.88 in a previous study [44], and, in this study, ranged from 0.66–0.87. Examples of items include: “Some students record or take photos of classmates with their mobile, to make fun”; and “Students hit classmates on the school campus”.

We used the Questionnaire of Experiences Related to Internet (CERI) prepared by Beranuy [44]. Specifically, this questionnaire is regarding experiences related to the related to problematic internet use in a survey made up of 10 items scored from range: 1 never/almost never, 4: almost always. The instrument consists of two factors: intrapersonal conflicts (INTRA) and interpersonal conflicts (INTER). The Cronbach’s α coefficients were 0.77 in INTRA and 0.75 in INTER in a previous study [45]. In this study, the Cronbach´s α coefficients were 0.7 in INTRA and 0.69 in INTER. Examples of items include: “Do you think that life without the internet is boring, empty and sad?”; and “When you are not connected to the internet, do you feel restless or worried?”.

### 2.3. Procedure

After obtaining approval from the ethics committee, the participating centers from the different geographic areas of the Region of Murcia were selected. A personal interview was arranged with the management team of the center as well as with the educational counselor to indicate the objectives of the study and facilitate participation. Subsequently, it was necessary to require the informed consent of the participants and the parents. Without them, the student’s results could not be included in the study. The instruments were administered in the selected classrooms during a 50-minute session. Anonymity, confidentiality, and voluntariness were maintained throughout the process.

### 2.4. Data Analysis

We used latent profile analysis to identify the subgroups [46]. The best model was chosen after analyzing the lowest values of AIC (Akaike Information Criterion) and the BIC (Bayesian Information Criterion), and also include 1% of the sample or more than 25 participants since small profiles should not be considered due to low statistical power (type II error), low power of generalization and are indicative of excessive extraction of profiles [47,48,49]. The groups were defined according to their intrapersonal (INTRA) and interpersonal conflicts (INTER) related to internet use of the Questionnaire of Experiences Related to Internet (CERI). Subsequently, analysis of variance (ANOVA) was used to examine the differences manifestations of school violence between the groups (Group 1, Group 2, Group 3 and Group 4) with the Bonferroni method. Cohen´s d test was used for the magnitude of the differences [50]. We used Mplus version 8 (Muthén & Muthén, Los Angeles, CA, USA) and Statistical Package for the Social Science version 23.0 (IBM Corp., Armonk, NY, USA).

### 2.5. Ethics Approval

The study protocols were approved by the Ethics Committee for Clinical Investigations of the University of Murcia (I.D. 2627/2019). The study was performed in accordance with the approved guidelines and the Declaration of Helsinki.

## 3. Results

In Table 1, Pearson’s correlations between the variables in the study are shown. They were significant and positive; therefore, we performed the latent profile.

**Figure 1:**
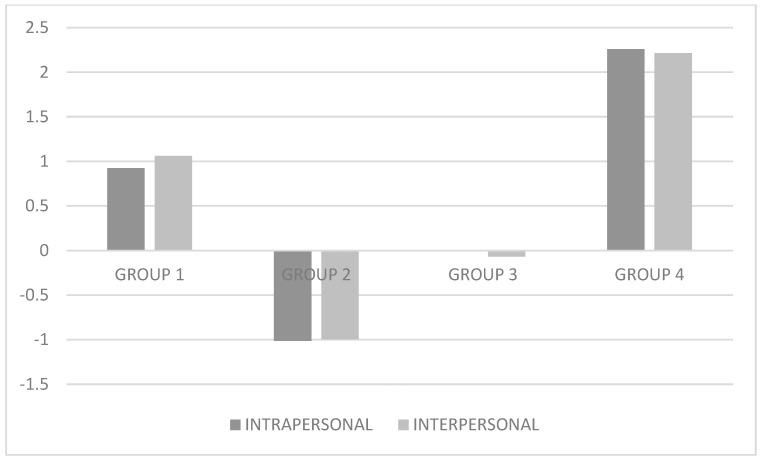
Graphical representation of the four-profiles. Group 1 (Problematic use), Group 2 (Non-problematic use), Group 3 (Moderate problematic use), and Group 4 (Severe problematic use).

Table 2 presents the models obtained (from two to seven classes). As the number of classes increases, the AIC, BIC and BIC-adjusted decreases, thus model 3 gives better values than model 2, model 4 better values than 3 and so on. However, the adjusted Vuong-Lo-Mendell-Rubin likelihood-ratio test and the adjusted Vuong-Lo-Mendell-Rubin likelihood-ratio test yielded a value of *p* > 0.05 for the 7-class model, so it was discarded. Models 5 and 6 had a class with less than 25 subjects so they were also discarded. Of the rest of the models, it is found that the 4-cluster model is the one with a lower BIC and AIC with an entropy of 0.72, which is still not the highest, is considered adequate, indicating that the 4 classes are capable of accurately classifying all the sample in 72%.The latent profile analysis, identified four different types of conflicts related to internet use: (a) a first group of 145 students characterized by high levels in intra- and interpersonal conflicts, called Problematic use; (b) a second group of 201 students characterized by low levels intra- and interpersonal conflicts, called Non-problematic use; (c) a third group of 433 students with moderate intra- and interpersonal conflicts, called Moderate problematic use; and (d) a fourth group of 31 students with very high levels in intra- and interpersonal conflicts, called Severe problematic use (see Figure 1).

Table 3 presents the results of the ANOVAs that revealed significant differences between four profiles for different types of conflicts related to internet use (intrapersonal and interpersonal) regarding the manifestations of school violence.

In Table 4, post hoc comparisons revealed that Group 4 (Severe problematic use) obtained significantly higher scores on the VTS, VPI, VPD, VVS, VVT, DB, and VICT compared with Group 1 (Problematic use), Group 3 (Moderate problematic used), and Group 2 (Non-problematic use). However, Group 1 (Problematic use) obtained significantly higher scores on the SE in comparison with Group 4 (Severe problematic use), Group 3 (Moderate problematic used), and Group 2 (Non-problematic use).

Similarly, post hoc comparisons revealed than Group 1 (Problematic use) obtained significantly higher scores on the VTS, VPI, VPD, VVS, VVT, SE, DB, and VICT compared with Group 3 (Moderate problematic used) and Group 2 (Non-problematic use).

Finally, post hoc comparisons revealed than Group 3 (Moderate problematic used) obtained significantly higher scores on the VTS, VPI, VPD, VVS, VVT, SE, DB, and VICT compared with Group 2 (Non-problematic use).

## 4. Discussion

This study allowed us to examine the existence of four profiles of different types of conflicts related to internet use (intrapersonal and interpersonal), similar to the four profiles of internet use (from non-problematic use to severe problematic use) evidenced in studies on problematic internet use [51]. Previous studies showed the existence of three clusters for problematic internet use on the CERI scale based on low, moderate, or high scores between the ages of 11 and 25 [22,43]. However, we showed four profiles primarily because we differentiated interpersonal and intrapersonal problems rather than taking them as a whole. In addition, we aimed to examine whether there were significant differences in bullying and cyberbullying among adolescents with a conflict related to internet use. In post hoc comparisons, the results revealed that Group 4 (Severe problematic use) is the most vulnerable group as it had higher values in all manifestations of school violence, with the exception of SE, unlike Group 1 (Problematic use), Group 2 (Non-problematic use), and Group 3 (Moderate problematic use). The SE was higher in Group 1 (Problematic use), which may be because these teens make inappropriate use of the internet due to the perception of social exclusion or rejections they perceive [41]. Group 3 had moderate values in all manifestations of school violence, and Group 1 was the group with the lowest values.

Therefore, of the teenagers in our study, the profiles with the most risk were the 31 students in Group 4 and 145 students in Group 1. These students have intrapersonal and interpersonal conflicts related to their use of the internet that, in turn, relate to greater perceptions of the different manifestations of school violence [41]. This is in line with studies that showed that the problematic use of technology has been linked to greater involvement in the different manifestations of school violence [36,37,41]. One could understand that the internet may be used in a problematic way as a way to alleviate aggression or prevent impulsivity. The lack of control in personal information is a strong predictor of cyberbullying, and, in turn, research demonstrated that cyberbullying coexists with traditional bullying [27,29,30,31,32,33,34,35].

Therefore, the results of the study are indicative that it is necessary to join forces from multidisciplinary teams to properly manage the problems associated with the problematic use of technology [29]. It is imperative to schedule preventive strategies focused on both the teen and the family and the school [15]. To do this, we consider it necessary to develop identity in a tight way in the adolescent stage to avoid this occurring at later educational stages, such as at university, and to continue to address problems in interpersonal relationships and abuse of the internet [26]. Similarly, it is necessary to promote self-esteem [23], coping strategies [52], emotional regulation [2,4,6,7,8,9,53], skills for conflict resolution and stress management, self-control, social and communication skills, and healthy leisure activities [16,19,20,54]. The family also has a relevant role to play in preventing internet addiction [6,14,18].

We also recommend the use of preventive programs, such as the ConRed cyberbullying program [55]. This program improves coping skills against cyberbullying. The program showed a reduction in information shared on the internet by victims, including a reduction in internet dependence by cyber-users and a reduction in traditional harassment. In cybervictims, there was a reduction in cybervictimization as well as traditional bullying. This indicates that programs that are effective against cyberbullying can also effectively combat traditional harassment. Similarly, another program that has shown its effectiveness in reducing the prevalence of aggression and cyberbullying as well as the abuse of the internet is the Asegúrate Program [56], with particular relevance for teachers [57]. Finally, the Cyberprogram 2.0 and the Cooperative Cybereduca 2.0 Videogame [58] or Program Prev@cib [59] were also shown to be effective in our country in reducing bullying and cyberbullying.

The limitations of the study include the potential for a cross-sectional study and longitudinal studies. Likewise, to investigate the use of other addictions, such as alcohol and other drugs [60], problematic use of the mobile phone, eating disorders, compulsive spending [61], anxiety [62] would be of interest that may be influencing the profiles found.

Finally, as the DSM-5 does not include internet addiction among the behavioral addictions, we consider it necessary to move forward in this direction to improve early detection to minimize the long-term effects and consequences and for intervention [61].

## 5. Conclusions

The use of technology in the adolescent stage has benefits and risks [2]. In this study, we examined the existence of four profiles of different types of conflict related to internet use (intrapersonal and interpersonal) regarding the manifestations of school violence. The results showed that students who appeared to have severe problematic use were the most involved in the different manifestations of school violence.

As the problematic use of the internet implies a significant impact on the well-being of a person in all fields (mental, physical, social, etc.) [29]; therefore, we must address the fact that the adolescent stage is a crucial stage in detecting the abusive uses of the internet that may end up being problematic [2,4,5]. Early detection and, above all, a comprehensive approach is essential [2]. In this sense, studies, such as this one, lay the foundations on the profiles of greatest risk and possible guidelines of action.

## Figures and Tables

**Table 1:** Pearson Correlation between the variables of study.

Variable	Intrapersonal	Interpersonal
VTS	0.395 **	0.381 **
VPI	0.281 **	0.332 **
VPD	0.303 **	0.281 **
VVS	0.254 **	0.275 **
VVT	0.216 **	0.202 **
SE	0.209 **	0.233 **
DB	0.102 **	0.186 **
VICT	0.288 **	0.296 **

Note: ** *p* < 0.01. VTS: violence of teachers towards students; VPI: physical indirect violence by students verbal; VPD: physical direct violence between students; VVS: violence among students; VVT: verbal violence of students towards teachers; SE: social exclusion; DB: disruptive behavior in the classroom; VICT: violence through information and communication technology.

**Table 2:** The fit of the all latent profiles models.

Models	AIC	BIC	BIC-Adjusted	LRT *p*	LRT-Adjusted	BLRT	Entropy	Size
2	4288.171	4321.051	4298.822	0.0000	0.0000	0.0000	0.726	0
3	4164.406	4211.376	4179.620	0.0181	0.0209	0.0000	0.708	0
**4**	**4121.276**	**4182.337**	**4141.055**	**0.0147**	**0.0171**	**0.0000**	**0.720**	**0**
5	4099.906	4175.059	4124.249	0.0009	0.0011	0.0000	0.768	1
6	4082.636	4171.880	4111.544	0.0000	0.0000	0.0000	0.731	1
7	4064.532	4167.866	4098.003	0.2709	0.2848	0.0000	0.745	1

Note: AIC = Akaike Information Criterion, BIC = Bayesian Information Criterion, LRT = Vuong-Lo-Mendell-Rubin likelihood-ratio test, BLRT = Bootstrap Likelihood Ratio test. Size: number of clusters with less than 25 subjects. Values in bold show the selected model.

**Table 3:** Means and standard deviations obtained by the profiles different types of intrapersonal and interpersonal and values of the eta^2^ parcial (*η_p_^2^*) for each variable of school violence.

Variable	Group 1		Group 2		Group 3		Group 4		Significance		
	*M*	*SD*	*M*	*SD*	*M*	*SD*	*M*	*SD*	*F_(2,212)_*	*p*	*η_p_^2^*
VTS	18.21	6.44	12.82	4.68	15.04	4.84	22.38	7.87	48.68	<0.001	0.15
VPI	7.54	2.92	5.77	2.13	6.45	2.37	8.77	3.01	23.31	<0.001	0.08
VPD	8.35	2.91	6.50	2.47	6.90	2.35	9.25	2.93	24.14	<0.001	0.08
VVS	12.32	3.66	10.18	3.00	11.59	3.37	13.96	3.19	19.13	<0.001	0.07
VVT	5.26	2.10	4.24	1.62	4.94	1.85	5.61	2.41	11.28	<0.001	0.04
SE	7.36	3.28	5.64	2.23	6.12	2.47	7.03	3.87	13.30	<0.001	0.05
DB	10.35	3.14	9.55	3.12	9.66	2.77	11.35	2.64	5.37	<0.001	0.02
VICT	11.67	6.03	8.47	3.37	9.46	4.08	13.41	7.35	21.60	<0.001	0.07

Note. Group 1 (Problematic use), Group 2 (Non-problematic use), and Group 3 (Moderate problematic used) and Group 4 (Severe problematic use). VTS: violence of teachers towards students; VPI: physical indirect violence by students verbal; VPD: physical direct violence between students; VVS: violence among students; VVT: verbal violence of students towards teachers; SE: social exclusion; DB: disruptive behavior in the classroom; VICT: violence through information and communication technology.

**Table 4:** Cohen´s d indexes for post- hoc contrast groups.

Variable	Group 1–Group 2	Group 1–Group 3	Group 1–Group 4	Group 2–Group 3	Group 2–Group 4	Group 3–Group 4
VTS	0.98 ***	0.60 ***	0.62 ***	0.46 ***	1.84 ***	1.44 ***
VPI	0.71 ***	0.43 ***		0.30 *	1.32 ***	0.96 ***
VPD	0.69 ***	0.58 ***			1.08 ***	0.98 ***
VVS	0.65 ***			0.43 ***	1.24 ***	0.71 **
VVT	0.56 ***			0.39 ***	0.79 ***	
SE	0.63 ***	0.46 ***			0.55 *	
DB					0.59 *	0.61 *
VICT	0.68 ***	0.48 ***			1.20 ***	0.90 ***

Note: * *p* < 0.05, ** *p* < 0.01, *** *p* < 0.01. Group 1 (Problematic use), Group 2 (Non-problematic use), and Group 3 (Moderate problematic used) and Group 4 (Severe problematic use). VTS: violence of teachers towards students; VPI: physical indirect violence by students verbal; VPD: physical direct violence between students; VVS: violence among students; VVT: verbal violence of students towards teachers; SE: social exclusion; DB: disruptive behavior in the classroom; VICT: violence through information and communication technology.
